# Aplasia Cutis Congenita of the Lower Limb: A Case Report

**DOI:** 10.7759/cureus.33376

**Published:** 2023-01-04

**Authors:** Abdulwahab M Alharthi, Abdulhadi A Turkistani, Bader Alallah, Jubara Alallah

**Affiliations:** 1 College of Medicine, King Saud Bin Abdulaziz University for Health Sciences, Jeddah, SAU; 2 Neonatology, King Saud Bin Abdulaziz University for Health Sciences, Jeddah, SAU; 3 Pediatric Neonatology, King Saud Bin Abdulaziz University for Health Sciences, Jeddah, SAU; 4 Pediatric Neonatology, King Khalid Medical City, National Guard Hospital, Jeddah, SAU

**Keywords:** congenital absence of skin, genetic, epidermolysis bullosa, bart's syndrome, aplasia cutis congenita

## Abstract

Aplasia cutis congenita type VI is a genetic disorder that presents with congenital skin absence, blistering, and nail abnormalities. We present the case of a male newborn who presented with an absence of skin in the entire left leg and the lower part of the left thigh. On the second day of life, he had new skin lesions that started to appear over the fingernail beds, nasal bridge, thighs, and buttocks. There were no other associated anomalies such as pyloric atresia, renal abnormalities, or ureteral stenosis. A diagnosis of Bart’s syndrome was made based on clinical diagnosis and previous presentation in the family. The patient developed sepsis and osteomyelitis of the lower limb and eventually died.

## Introduction

Bart’s syndrome is a rare genetic condition that presents with congenital skin absence, blistering, and nail abnormalities. It is also called aplasia cutis congenita type VI [1]. Aplasia cutis congenita (ACC) is inherited in the autosomal dominant form with complete penetrance but variable expression. It is a rare disorder with an incidence of one to three in 10,000 live births approximately [2]. In most cases, ACC is usually an isolated finding, however, it can be linked to a variety of genetic disorders and congenital anomalies [3]. The pathophysiology of ACC is not fully understood, ACC is characterized by the congenital absence of the skin anywhere on the body, but most cases present with scalp involvement [4].

In 1966, the first case of Bart’s syndrome was discovered, which describes the association between ACC and epidermolysis bullosa (EB) [5]. Furthermore, Bart’s syndrome criteria involve localized widespread congenital skin absence, blistering, and nail abnormalities. It has been found that ACC is connected with all forms of EB, i.e., simplex type, junctional type, or dystrophic type [6].

In this case, we report a rare case of a full-term infant who presented with ACC at birth and on the second day of life, developed features consistent with EB.

## Case presentation

A male newborn presented with an absence of skin on the whole left leg and the lower part of the left thigh. He was born at a gestational age of 38 weeks + 5 days with a birthweight of 2.320 kg delivered by spontaneous vaginal delivery to a booked mother. She was gravida 5 para 4 with no medical history but with a history of two previous siblings who died in infancy due to epidermolysis bullosa. The second sibling was delivered in our hospital and was diagnosed by skin biopsy to have a junctional type of epidermolysis bullosa.

**Figure 1:**
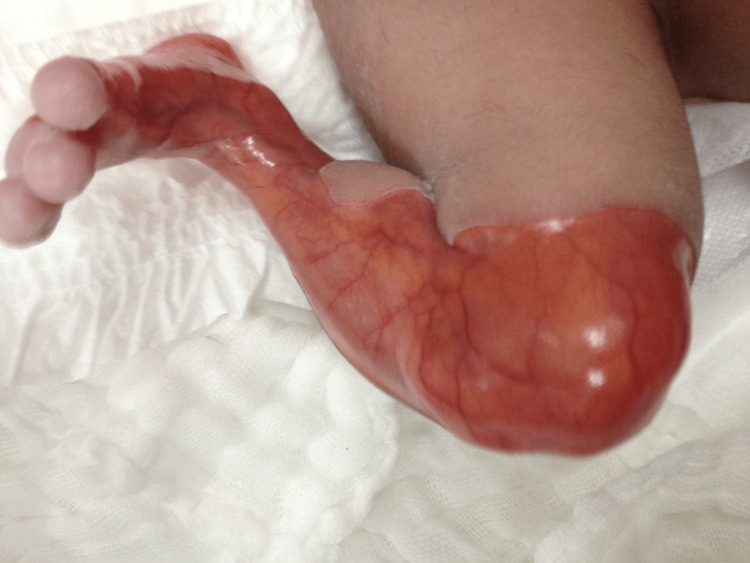
Absence of the skin of the left lower limb from the dorsum of the foot extended to above the knee with a clear demarcation line and normal skin appearance of the rest of the leg

**Figure 2:**
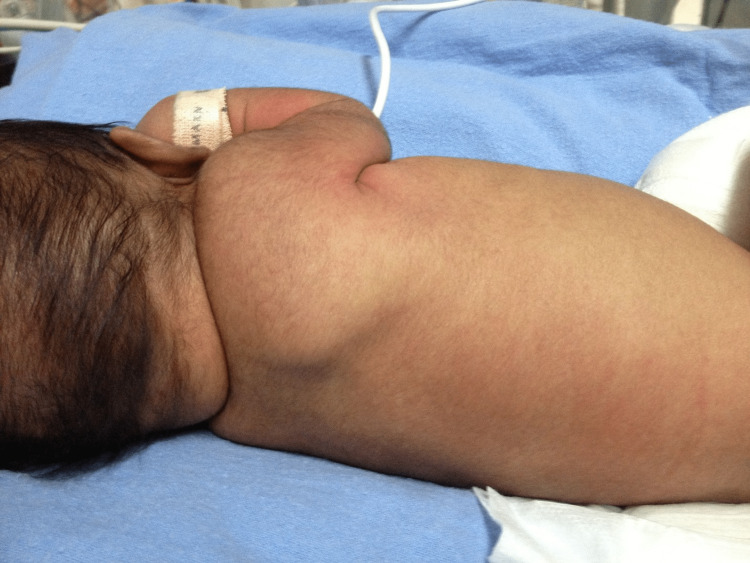
All parts of the body after birth were normal with no lesions

The parents are first-degree relatives with two normal, living kids. The delivery was uneventful and the neonatal intensive care unit team was called to assess the baby. The baby had aplasia cutis congenita of the whole left leg and the lower part of the left thigh (Figure 1). The rest of the body, including fingers, nails, and hair, was not affected (Figure 2). The baby was transferred to the neonatal intensive care unit (NICU) for further management.

**Figure 3:**
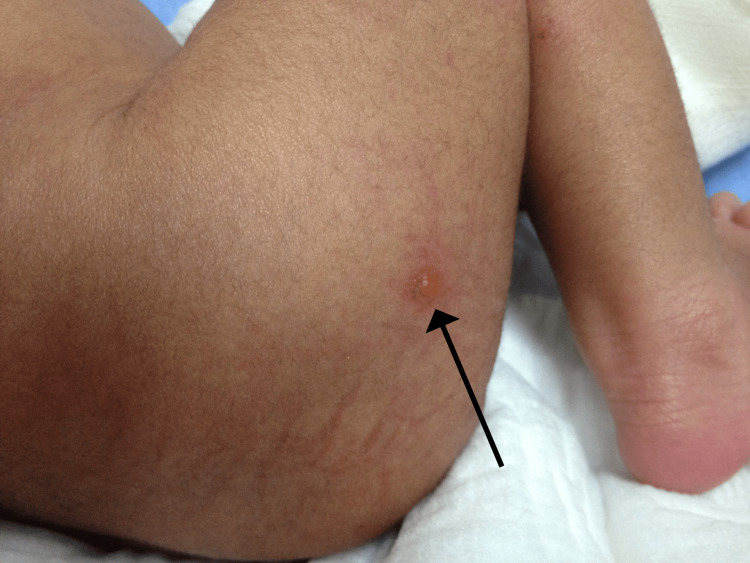
A new skin lesion (blister) appeared on the second day of life

Dermatology, plastic surgery, and genetic consultations were done. The plastic surgery team had the impression of aplasia cutis congenita, and they recommended wound dressing twice daily with Fucidin, Xeroform, and dry gauze. The genetic team had the impression of epidermolysis bullosa based on the clinical presentation and family history of two siblings with the same condition and one confirmed skin biopsy. The dermatology team agreed with the diagnosis of epidermolysis bullosa and recommended continuing wound dressing and avoiding trauma; otherwise, the baby was clinically stable. He had new skin lesions that started to appear on the second day of life over the fingernail beds, nasal bridge, thighs, and buttocks (Figure 3).

The baby was discharged after 25 days in the NICU with a weight of 2.630 kg, in stable condition, and feeding well, on supportive treatment with wound dressing and regular wound care clinic weekly after discharge.

At three months old, he presented with a left lower limb infection and toxic shock syndrome, and osteomyelitis was suspected. There was a foul-smelling discharge, and bluish discoloration of the left lower limb. The patient was then admitted to the pediatric intensive care unit in critical condition. Blood cultures showed *Staphylococcus aureus*. Repeated blood cultures showed* Staphylococcus aureus* and wound cultures showed *Pseudomonas* and *Proteus *species. He was on Amphotericin B, Tazocin, and Meropenem.

He had a lower limb X-ray, which showed a slight discrepancy in the length of the bone, which was more on the left side with poor development of metaphysis and epiphysis on the left side and periosteal reaction at the distal femur. There was a suspicion of osteomyelitis but the patient could not undergo a CT scan because of his condition, and he was treated clinically as osteomyelitis. The patient died 42 days later.

## Discussion

ACC is a rare group of heterogeneous diseases constituting a failure of the skin to fully develop. The exact mechanism is not yet fully understood. One hypothesis states that it is due to the incomplete closure of the neural tube during embryonic development. Other proposed theories are intrauterine trauma, placental insufficiency causing vascular compromise, intrauterine infections, genetic mutations, and teratogens [2,3].

Also, as a form of EB, the problem lies in ultrastructure anchoring fibrils. The defect is a problem of attachment between or within the layers of skin. Loss or diminished function of C7 leads to weakness in the structural architecture of the dermal-epidermal junction and mucosal membranes.

The main clinical features of ACC are localized widespread congenital skin absence, blistering, and nail abnormalities [7]. However, in more severe cases, ACC can present with other congenital anomalies such as pyloric atresia, ureteral stenosis, and renal abnormalities [8]. In our case, there was no association with any other abnormality.

Bart’s syndrome is usually diagnosed based on clinical features, but in some cases, a skin biopsy might be needed to determine the type of epidermolysis bullosa [9]. In our case, the diagnosis of Bart’s syndrome was based on clinical features and previous confirmation of EB by skin biopsy in the family.

Our case was managed conservatively with wound care as per protocol. The prognosis of Bart’s syndrome is usually good. It depends on the severity and extent of ACC, epidermolysis bullosa subtype, accompanying abnormalities, and therapy success, all of which influence the outcome of Bart syndrome [9]. The estimated mortality rate is 20% to 55% and this is mainly due to complications such as infection of the skin lesions and sagittal sinus bleeding when the lesion is around the scalp [10]. In our case, the patient developed multiple complications and died unfortunately due to infection.

## Conclusions

Aplasia cutis congenita is a rare congenital disorder characterized by the absence of skin anywhere on the body. In most cases, ACC is usually an isolated finding, however, it can be linked to a variety of genetic disorders and congenital anomalies like in our case. Usually, epidermolysis bullosa blisters are noticed at birth, but it's not uncommon to appear later; like in our case. the baby was born with cutis aplasia of the left lower limb but the blisters appeared on the second day, with a high index of suspicion of EB based on the strong family history.

Although the prognosis is widely varied depending on the complications that develop, such a disease can be fatal. Early identification and management are required to achieve the best outcome and avoid complications.
